# Evaluation of the virucidal efficacy of Klaran UVC LEDs against surface-dried norovirus

**DOI:** 10.1099/acmi.0.000323

**Published:** 2022-01-12

**Authors:** Richard M. Mariita, Amy C. Wilson Miller, Rajul V. Randive

**Affiliations:** ^1^​ Crystal IS, Inc., an Asahi Kasei company, 70 Cohoes Avenue, Green Island, New York, 12183, USA

**Keywords:** *Caliciviridae*, feline calicivirus, inactivation, norovirus, surrogate, UVC

## Abstract

Human norovirus (HuNoV) is a highly contagious pathogenic virus that is transmitted through contaminated food, water, high-touch surfaces and aerosols. Globally, there are an estimated 685 million infections annually due to norovirus, including 200 million affecting children under the age of 5. HuNoV causes approximately 50, 000 child deaths per year and costs an estimated USD $60 billion annually in healthcare. This study sought to determine the inactivation profile of ultraviolet subtype C (UVC) against norovirus using a UVC light-emitting diode (LED) array, KL265-50V-SM-WD. The array emitted radiation at 269 nm peak wavelength and a measured fluence of 1.25 mW cm^−2^ at a 7 cm source–surface distance. Since the HuNoV is not cultivable, the study utilized feline calicivirus (FCV) ATCC VR-782, a recommended surrogate as challenge organism. The test followed modified ASTM E2197. Assessment of virus inactivation was performed using a plaque assay method. With irradiance at a UVC dose of 22.5 mJ cm^−2^, the study obtained 99.9 % virus reduction (3 log reduction). The results demonstrate that the UVC LED array can provide effective inactivation of HuNoV.

## Introduction

Human norovirus (HuNoV), formerly known as Norwalk virus, is an RNA virus belonging to the family *Caliciviridae* [1]. This is a highly contagious, small, non-enveloped enteric pathogen that is transmitted through person-to-person contact and unsanitary food handling [2], contaminated water and high-touch surfaces [1], and can also be spread via aerosols [3]. Because it only requires a small inoculum to produce an infection (<100 viral particles), is pathogenic and has the ability to survive in different environments, HuNoV is responsible for substantial comorbidity, especially in healthcare and community settings [1], such as daycare centres, nursing homes, hospital wards, schools, restaurants, catered events and cruise ships [4].

Globally, there are an estimated 685 million cases of norovirus infections annually, with ~200 million of them being among children under 5 years old, leading to an estimated 50, 000 child deaths and healthcare costs estimated at USD $60 billion per year [5]. In the USA, HuNoV is associated with 80–90 % of the reported outbreaks and is the leading cause of nonbacterial gastroenteritis [4]. On average, in the USA, HuNoV causes an average of 570–800 deaths, 56, 000–71, 000 hospitalizations, 400, 000 ER visits, 1.7–1.9 million outpatient visits and 19–21 million total illnesses per year [6]. Outbreaks involve people in high-risk groups, particularly young children under 5 years of age, the elderly above 65 [6], travellers, soldiers and the immunocompromised [4]. To compound the problem, only a limited number of disinfectants are effective against HuNoV has at present [7]. Unlike with other viral pathogens, such as severe acute respiratory syndrome coronavirus 2 (SARS-CoV-2) [8], vaccine design and production efforts against HuNoV have not been successful. Study of HuNoV has further been hindered by the inability to propagate it in cell cultures [9]. Because of this, another virus from the family *Caliciviridae*, feline calicivirus (FCV), is frequently used as a surrogate [10], especially in determining the virucidal efficacy of disinfectants [7].

For RNA viruses, ultraviolet subtype C (UVC) induces inactivation that leads to RNA damage [11], the principal factor in loss of viral infectivity [12], while FCV is thought to be a reasonable surrogate for HuNoV UVC inactivation profiles. Light-emitting diodes (LEDs) made from semiconductor materials can be used to produce UVC in the range of 200–280 nm [13], which is considered to be germicidal [14]. LEDs emitting UVC have been used in agriculture, water and the food industry for microbial inactivation because they have several advantages over conventional sources [13]. Such advantages include compact size that makes incorporation easier, being nonhazardous (no health hazards due to the possibility of mercury contamination), showing high performance and having a long lifetime [13].

The inactivation of HuNoV is a major focus in water purification [15]. This is because of the existing economic and public burden associated with HuNoV, thus there is a need for additional appropriate interventions, including effective inactivation strategies. Here, the study utilized a disinfection assay to investigate the virucidal activity of a UVC LED array (KL265-50V-SM-WD) against FCV ATCC VR-782 on magnetic stainless steel discs. Steel coupons were used because human norovirus transmission can be via contaminated surfaces (fomites) [12]. The aim of the study was to determine the inactivation profile of a UVC LED array with peak emission of 269 nm against norovirus.

## Methods

### Electrical measurements of the UVC LED array

A USB4000 photospectrometer (Ocean Optics) was used to confirm the emitted radiation peak wavelength of the UVC LED array. For UVC dose, confirmation was achieved using the X1 handheld optometer (Gigahertz-Optik). The UVC LED array tested in this study was KL265-50V-SM-WD, which is rated between 70–80 mW at 500 mA and was driven at 350 mA, yielding an expected 56–64 mW at beginning of life.

### Experimental set-up

The disinfection assay was carried out using Eagle’s modified medium with 2 % fetal bovine serum (FBS) as the test solution. The virus was applied on 20 mm diameter magnetic stainless steel discs, spread and dried at room temperature prior to exposure to UVC. The sample was put in the centre of the box, directly opposite and aligned to the light source, where maximum intensity is found. The distance between the LED and microbe was fixed at 7 cm.

### UVC disinfection procedure

The inactivation experiments were performed in duplicate per irradiation period using feline calicivirus (FCV) ATCC VR-782 spread on stainless steel magnetic discs following a modified ASTM E2197: Standard Quantitative Disk Carrier Test Method [16]. Specifically, this was done by applying 32 µl of standardized FCV on each disc, spreading to within 1 mm of the edges, and then air drying at room temperature. The stainless steel magnetic discs were then irradiated for 12, 18 and 22 s prior to suspension in 10 ml modified SCDLP (soybean, casein digest agar with lecithin and polysorbate) medium for virus recovery. SCDLP buffer was modified by having the formulation without lecithin or polysorbate 80. Additionally, 0.1 % bovine serum albumin was added as a stabilizing solution and 1× penicillin–streptomycin antibiotics was added to inhibit bacterial contamination of cell cultures.

### Quantitation of viral particles

To quantify viable viral particles, the dilution plate method was followed, with Crandell–Rees feline kidney (CRFK) ATCC CCL-94 cell lines being used for growth [17]. Viral particles were determined using crystal violet for staining to enable visualization of plaques for counting, giving plaque-forming units (p.f.u.) [18]. Mean p.f.u. between controls and irradiated samples were then used to calculate reduction following the formula:

$$log\;reduction=log_{10}\left(\frac{A}{B}\right)$$

where *A*=p.f.u./disc for control (no irradiation with UVC) and *B*=p.f.u./disc for UVC ON at a given irradiation period in seconds.

## Results and discussion

**Fig. 1.**
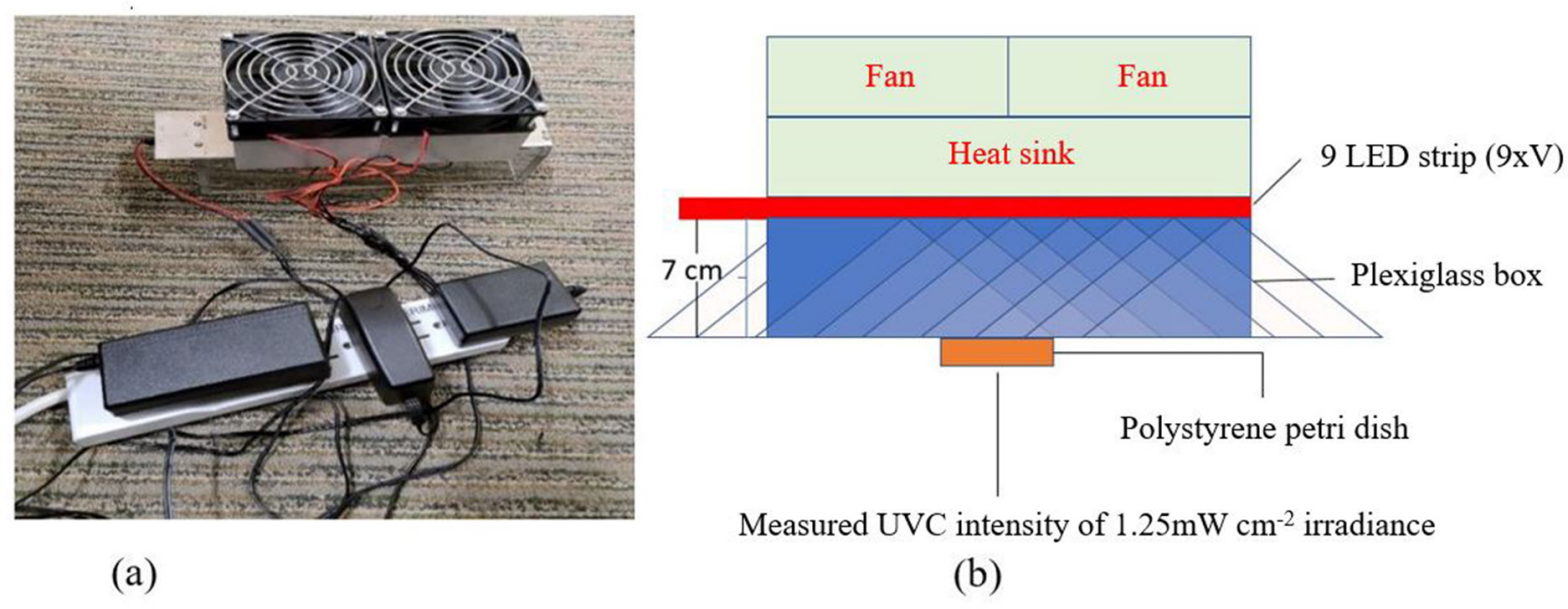
(**a**) The UVC array was driven at 350 mA during the norovirus inactivation study. (**b**) The array had two fans and a heat sink for thermal management and was tested at 7 cm height.

The confirmed peak wavelength of the UVC array was 269 nm, and at 7 cm height (Fig. 1) it obtained an intensity of 1.25 mW cm^−2^. At 12 s and a dose of 15 mJ cm^−2^, the LED array obtained a 2.70 log reduction of viable FCV virus (Table 1). At 18 s or more (22.5 mJ cm^−2^ or higher dose), a >3 log reduction of viable FCV virus was obtained. No inactivation differences at 18 and 22 s (22.5 and 27.5 mJ cm^−2^) of irradiation were revealed (Table 1). The experiment were performed in duplicate (intra-experimental repeats). The exhibited virucidal activity against FCV suspensions air dried on stainless steel magnetic discs is promising because surfaces are vectors of HuNoV transmission during outbreaks [19].

**Table 1.** Inactivation performance of an array (KL265-50V-SM-WD) emitting UVC radiation at 269 nm peak wavelength with intensity of 1.25 mW cm^−2^ revealed no inactivation differences at 18 and 22 s of irradiation. A dose of 22.5 mJ cm^−2^ was enough to obtain a >3 log reduction. All experiments were performed in duplicate (intra-experimental repeats) and means were used for inactivation efficacy determination

Exposure time (s)	Dose (mJ cm^−2^)	Average viral burden (p.f.u./disc)	Average log viral burden (p.f.u./disc)	Log reduction compared to control (0 s)	Percentage reduction
Zero (control)	0.0	4.00+05	5.60	na	na
12	15.0	8.00+02	2.90	2.70	99.80
18	22.5	1.50+02	2.18	3.43	99.96
22	27.5	2.00+02	2.30	3.30	99.95

na, not applicable.

These results are specific to 269 nm against FCV, the commonly used HuNoV model organism. Although the spectral sensitivity of HuNoV has not been investigated, it should be expected that different wavelengths will perform differently against viral pathogens, even with the same dose [20]. In coronaviruses for instance, a study by Gerchman *et al*., [20] demonstrated that UVC LEDs emitting radiation at peak wavelength between 267 and 279 nm were more effective at inactivation. A similar approach, however, should be utilized to confirm performance against HuNoV so as to determine sensitivities. Previously, investigations using UV_254_, a commonly used disinfection process in the water industry, established an inactivation rate constant for HuNoV [15]. Such an approach should be utilized in future studies with other UVC LEDs with different peak emissions to enable sensitivity comparisons against other surrogates.

The findings from the current study demonstrate the potential application of KL265-50V-SM-WD UVC arrays in cruise ships and resorts, especially living and dining quarters, where there are high risks of HuNoV acquisition, which can lead to disease outbreaks [21]. With necessary radiation safety considerations, results can further be applied in other areas of close living quarters or with shared dining facilities to help disrupt viral transmission [1]. Such areas include those that have reported norovirus outbreaks, such as schools [22], military training centres and fields of operation [23], healthcare facilities [24] and the restaurant and catering industry [25], as well as municipal and industrial water systems [26].

## Conclusions

In this study, a 22.5 mJ cm^−2^ UVC dose was found to be sufficient to achieve a >3 log reduction against FCV. The use of UVC LEDs thus promises a reduction of virus transmission during outbreaks. These findings demonstrated that UVC LEDs could serve as an effective and rapid tool in the fight against human norovirus by preventing spread via fomites.

### Availability of data and material

The original laboratory report with data is available upon request.
